# Survival of patients with orbital and eyelid rhabdomyosarcoma treated on Children’s Oncology Group studies from 1997 to 2013: A report from the Children’s Oncology Group

**DOI:** 10.1002/cncr.34723

**Published:** 2023-03-01

**Authors:** Jonathan Metts, Wei Xue, Zhengya Gao, Ralph Ermoian, Julie A. Bradley, Michael A. Arnold, Roshni Dasgupta, Rajkumar Venkatramani, David Walterhouse

**Affiliations:** 1 Cancer and Blood Disorders Institute, Johns Hopkins All Children’s Hospital, St Petersburg, Florida, USA; 2 Department of Biostatistics, College of Public Health and Health Professions and College of Medicine, University of Florida, Gainesville, Florida, USA; 3 Department of Radiation Oncology, University of Washington, Seattle, Washington, USA; 4 Department of Radiation Oncology, University of Florida College of Medicine, Jacksonville, Florida, USA; 5 Department of Pathology and Laboratory Medicine, Children’s Hospital Colorado, Anschutz Medical Campus, Aurora, Colorado, USA; 6 Department of Pathology, University of Colorado, Anschutz Medical Campus, Aurora, Colorado, USA; 7 Division of Pediatric General and Thoracic Surgery, Cincinnati Children’s Hospital Medical Center, University of Cincinnati, Cincinnati, Ohio, USA; 8 Department of Pediatrics, Texas Children’s Hospital, Baylor College of Medicine, Houston, Texas, USA; 9 Department of Pediatrics, Ann & Robert H. Lurie Children’s Hospital of Chicago, Northwestern University Feinberg School of Medicine, Chicago, Illinois, USA

**Keywords:** orbital, pediatric, rhabdomyosarcoma, sarcoma, survival

## Abstract

**Background:**

Orbital rhabdomyosarcoma (ORMS) commonly presents as low-risk disease (stage 1, group I-III, embryonal RMS) with excellent outcome. Long-term follow-up of patients with low-risk ORMS and outcomes of less common subgroups of ORMS treated on recent Children’s Oncology Group (COG) trials have not been reported.

**Methods:**

Patients with ORMS enrolled on COG trials from 1997 to 2013 were identified. Demographic information and disease characteristics were collected. Outcomes were determined for the following subgroups: 1) low-risk ORMS, 2) resected (group I/II) low-risk ORMS, 3) non-low-risk ORMS, and 4) recurrent ORMS. Event-free survival (EFS) and overall survival (OS) were estimated using the Kaplan-Meier method.

**Results:**

The authors identified 218 patients with ORMS. Most tumors were embryonal/botryoid (*n* = 169; 77.5%), <5 cm (*n* = 213; 97.7%), group III (*n* = 170; 78.0%), and without lymph node involvement (N0; *n* = 215; 98.6%). For 192 patients with low-risk ORMS, the 10-year EFS and OS rates were 85.5% (95% confidence interval [CI], 77.0%-94.0%) and 95.6% (95% CI, 90.8%-100.0%), respectively. Those with group I/II low-risk ORMS (*n* = 5 in group I; *n* = 39 in group IIA) had 10-year EFS and OS rates of 88.0% (95% CI, 72.6%-100.0%) and 97.6% (95% CI, 90.0%-100.0%), respectively. Twenty-six patients with non-low-risk ORMS had 5-year EFS and OS rates of 88.5% (95% CI, 75.6%-100.0%) and 95.8% (95% CI, 87.7%-100.0%), respectively. For patients with recurrent ORMS, the 10-year OS rate from the time of recurrence was 69.4% (95% CI, 50.0%-88.8%).

**Conclusions:**

Patients with ORMS had favorable long-term survival outcomes on COG studies from 1997 to 2013, including those who had both low-risk and non-low-risk disease. A significant proportion of patients with recurrent ORMS may achieve long-term survival.

## INTRODUCTION

Orbital rhabdomyosarcoma (ORMS) comprises 10% of all rhabdomyosarcoma (RMS). The orbit is designated a favorable primary site by the Intergroup Rhabdomyosarcoma Study Group (IRSG) modified tumor, node, metastasis (TNM) staging system.^1–3^ The standard treatment approach to ORMS on IRSG and subsequent Children’s Oncology Group (COG) studies has been biopsy followed by chemotherapy and radiotherapy, so that, at presentation, most patients have group III disease.^4^ The highest failure-free survival (FFS) rate reported for patients with group III ORMS occurred on Intergroup Rhabdomyosarcoma Study IV (IRS-IV) (3-year FFS, 94%; overall survival [OS], 98%); however, intensive therapy was administered with 26.4 g/m^2^ cyclophosphamide or 126 g/m^2^ ifosfamide and 50.4-59.4 Gray (Gy) of radiation, increasing the risks of long-term morbidity.^5^

Beginning in 1997, COG trials stratified patients by risk group, and the majority of those with ORMS (nonalveolar; groups I-III) were included on the subsequent low-risk studies D9602 (ClinicalTrials.gov identifier NCT00002995) and ARST0331 (ClinicalTrials.gov identifier NCT00075582).^6^ These noninferiority treatment-reduction trials reported 5-year FFS and OS rates of 86% and 96%, respectively, on D9602 (group III ORMS only) and 3-year FFS and OS rates of 86% and 97%, respectively, on ARST0331 (group I-III ORMS).^4,7^ Although FFS with these approaches fell below 90%, OS remained >95% with less alkylator exposure (0 g/m^2^ cyclophosphamide on D9602 and 4.8 g/m^2^ cyclophosphamide on ARST0331) and reduced radiation dosing for patients with group IIA (36 vs. 41.4 Gy on IRS-IV) and group III (45 vs. 50.4-59.4 Gy on IRS-IV) orbit disease.^4,7–9^ The balance between FFS, OS, and toxicity was believed to favor the low-risk therapies over IRS-IV based on these 3-year to 5-year outcome results. However, long-term survival outcomes have not been reported for ORMS on these trials. Specifically, it is important to determine whether the favorable survival rates on D9602 and ARST0331, despite significant therapy reductions for group III disease from IRS-IV, were preserved over time.

Outcomes for less common subgroups of ORMS, including group I/II low-risk disease and ORMS treated on intermediate-risk and high-risk COG trials from 1997 to 2013, also deserve investigation. Beginning with D9602 and continuing through ARST0331, the recommended radiation dose for group IIA RMS decreased from 41.4 Gy on IRS-IV to 36 Gy; however, survival outcomes specifically for group I/II low-risk ORMS were not previously reported from D9602 and ARST0331. In addition, although survival for patients with alveolar ORMS on IRS I-IV studies was found to be inferior to that for patients with embryonal ORMS, the outcomes of patients with alveolar and/or metastatic ORMS (non-low-risk ORMS) on risk-group-stratified COG trials conducted since 1997 have not been reported.^10^ Finally, the outcome of patients with recurrent ORMS was retrospectively reported from IRS-III/IRS-IV but has not been reported from more recent COG trials.^11^ Therefore, the objectives of this study were to determine the long-term survival outcome of patients with (1) low-risk ORMS treated on COG trials, including the subgroup of patients with resected, low-risk ORMS (group I/II); (2) non–low-risk ORMS; and (3) recurrent ORMS.

## MATERIALS AND METHODS

### Patient selection

Patients with ORMS, defined as having a primary tumor subsite of the orbit or eyelid, were identified from all risk-group–stratified COG RMS trials for newly diagnosed patients with published primary articles at the time of this analysis (D9602, D9802 [ClinicalTrials.gov identifier NCT00003955], D9803 [ClinicalTrials.gov identifier NCT00003958], ARST0331, ARST0431 [ClinicalTrials.gov identifier NCT00354744], ARST0531[ClinicalTrials.gov identifier NCT00354835], and ARST08P1[ClinicalTrials.gov identifier NCT01055314]; see Table S1 for study descriptions).^4,7,12–16^ Informed consent/assent from the patient and/or parent/guardian was obtained before enrollment, and all trials were approved by the institutional review board of participating institutions or by the Pediatric Central Institutional Review Board of the National Cancer Institute. Demographic information and disease characteristics, including histology, clinical group, tumor size, tumor invasiveness, clinical nodal status, *FOXO1* fusion status (when available), disease response, and recurrence site (when applicable), were collected. Because of evolving definitions of RMS histologies over time, it is important to note that all histologies presented here are based on central re-review classification using current definitions.^17^ Outcomes were estimated for the following groups: (1) low-risk ORMS treated on D9602 and ARST0331; (2) resected (group I/II), low-risk ORMS treated on D9602 and ARST0331; (3) non–low-risk ORMS, defined as ORMS enrolling on intermediate-risk and high-risk trials D9802, D9803, ARST0431, ARST0531, and ARST08P1; and (4) recurrent disease, defined as disease progression or disease relapse, on all trials.

### Statistical analysis

Demographics and disease characteristics were summarized descriptively. Event-free survival (EFS) was defined as the time from the start of treatment to the first occurrence of disease progression, recurrence, second malignancy, or death. OS was defined as the time from the start of treatment to death from any cause, except for OS for recurrent ORMS, which was defined as the time from first documented recurrence to death from any cause. Median follow-up was based only on censored times. The Kaplan–Meier method was used to estimate survival distributions, and the Peto–Peto method was used to estimate the standard error of the Kaplan–Meier estimate. EFS and OS were estimated at 10 years for low-risk ORMS, group I/II low-risk ORMS, and recurrent ORMS and at 5 years for non–low-risk ORMS because of the shorter duration of follow-up for patients enrolled on ARST0531. The analysis was based on the data frozen on June 30, 2018.

## RESULTS

We identified 218 patients with ORMS who were enrolled on COG trials from 1997 to 2013 (Table 1). Most patients were enrolled on low-risk studies D9602 and ARST0331 (*n* = 192; 88.1%). The majority of patients were male (*n* = 129; 59.2%) and were aged 1–9 years (*n* = 159; 72.9%). Twenty-one patients had an eyelid subsite (9.6%). Consistent with prior reports, most tumors were classified as embryonal/botryoid histology (*n* = 169; 77.5%), group III (*n* = 170; 78.0%), noninvasive (T1; *n* = 211; 96.8%), and without clinical regional lymph node involvement (N0; *n* = 215; 98.6%). A single patient with clinical nodal disease (N1) was noted: this patient had alveolar histology, had *PAX3-FOXO1* fusion, and was alive without recurrence at last follow-up. Fusion status was available for 26 patients (all histologies); 13 were fusion-positive (11 *PAX3-FOXO1*, two *PAX7-FOXO1*), and 13 were fusion-negative. All fusion-positive patients had alveolar histology except one with histology *not otherwise specified*. One eyelid tumor was fusion-positive (*PAX7-FOXO1*). Among 19 patients with alveolar histology who had available fusion status, 12 were fusion-positive (11 *PAX3-FOXO1*, one *PAX7-FOXO1*), and seven were fusion-negative.

Low-risk trials (D9602 and ARST0331) enrolled 192 patients with a median follow up of 8.7 years. Most had embryonal/botryoid histology (*n* = 166; 86.5%; Table 1). Six low-risk patients had alveolar histology; these were enrolled on D9602 before an amendment in 1999 excluding alveolar histology from the trial. By clinical group, 148 patients had group III disease (77.1%), there were five in group I (2.6%) and 39 in group IIA (20.3%). There were 25 ORMS recurrences on low-risk trials; all were local.

**FIGURE 1:**
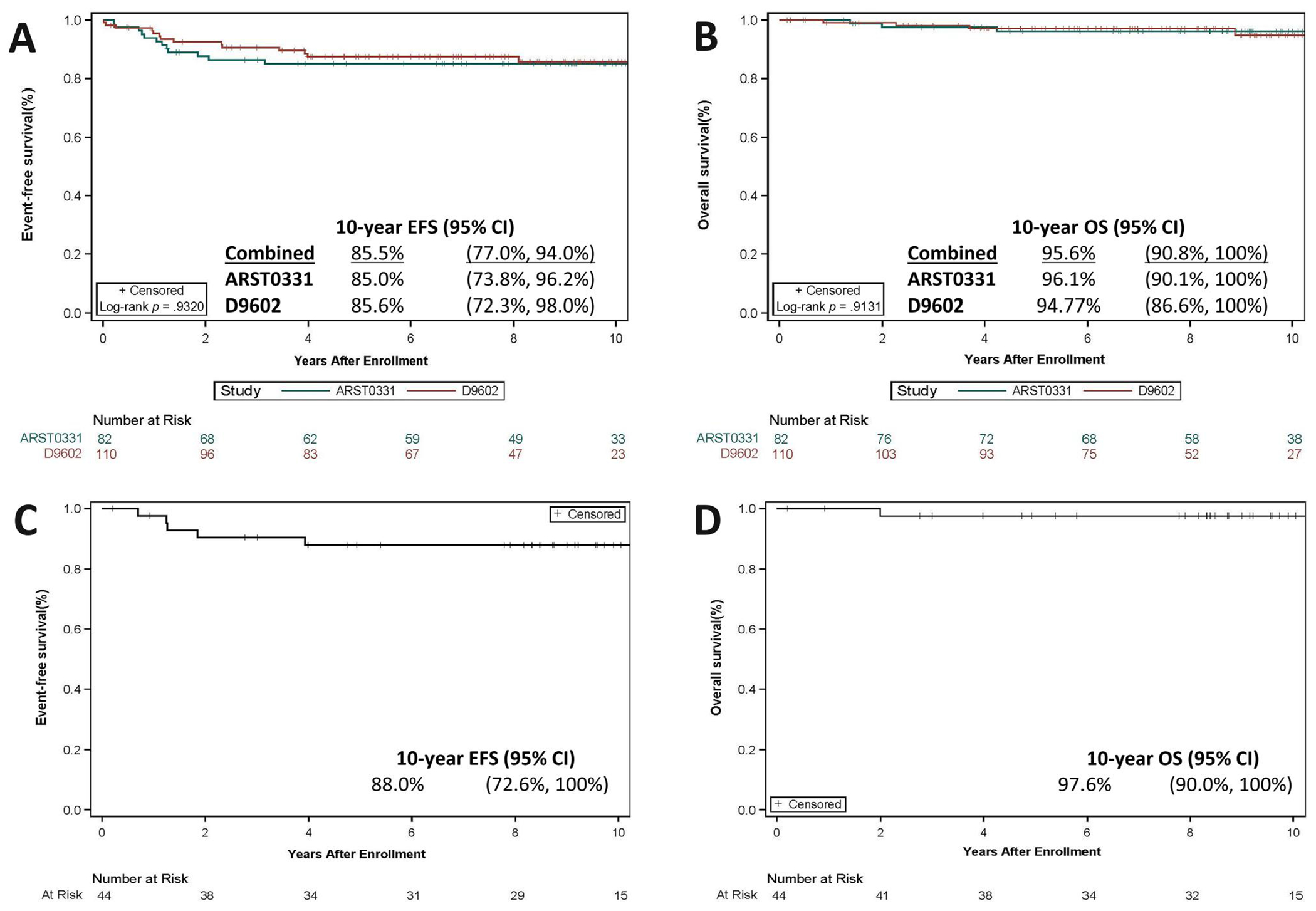
Kaplan–Meier distribution for low-risk orbital rhabdomyosarcoma. (A) EFS and (B) OS are illustrated for COG trials D9602 (red) and ARST0331 (green). (C) EFS and (D) OS are illustrated for patients with group I/IIA low-risk orbital rhabdomyosarcoma. Estimated 10-year EFS and OS rates for each Kaplan–Meier curve are indicated at the bottom right in each graph. CI indicates confidence interval; COG, Children’s Oncology Group; EFS, event-free survival; OS, overall survival.

The 10-year EFS and OS rates for ORMS on low-risk trials was 85.5% (95% confidence interval [CI], 77.0%-94.0%) and 95.6% (95% CI, 90.8%-100.0%), respectively (Figure 1A,B). Three second neoplasms occurred in two patients after 5 years; all developed in patients with group III embryonal ORMS on D9602 who received radiation therapy, including an osteosarcoma of the cheek and anaplastic astrocytoma of the brain in a patient 8 years after enrollment, and a mucoepidermoid carcinoma of the tongue in another patient 11 years after enrollment.

Forty-four patients were enrolled on low-risk trials with group I (*n* = 5) and group II (*n* = 39, all group IIA) ORMS and had a median follow-up of 9.2 years (Table 1). Eleven of these were eyelid tumors (two group I, nine group IIA), the majority were <3 cm in size (*n* = 35; 79.6%), and most had embryonal/botryoid histology (*n* = 40; 90.9%). There were five recurrences; all were local (four were originally group IIA, one was group I). The 10-year EFS and OS for group I and IIA patients was 88.0% (95% CI, 72.6%-100.0%) and 97.6% (95% CI, 90.0%-100.0%), respectively (Figure 1C,D). The group IIA subgroup had 10-year EFS and OS rates of 89.1% (95% CI, 72.4%-100.0%) and 97.3% (95% CI, 88.3%-100.0%), respectively.

**FIGURE 2:**
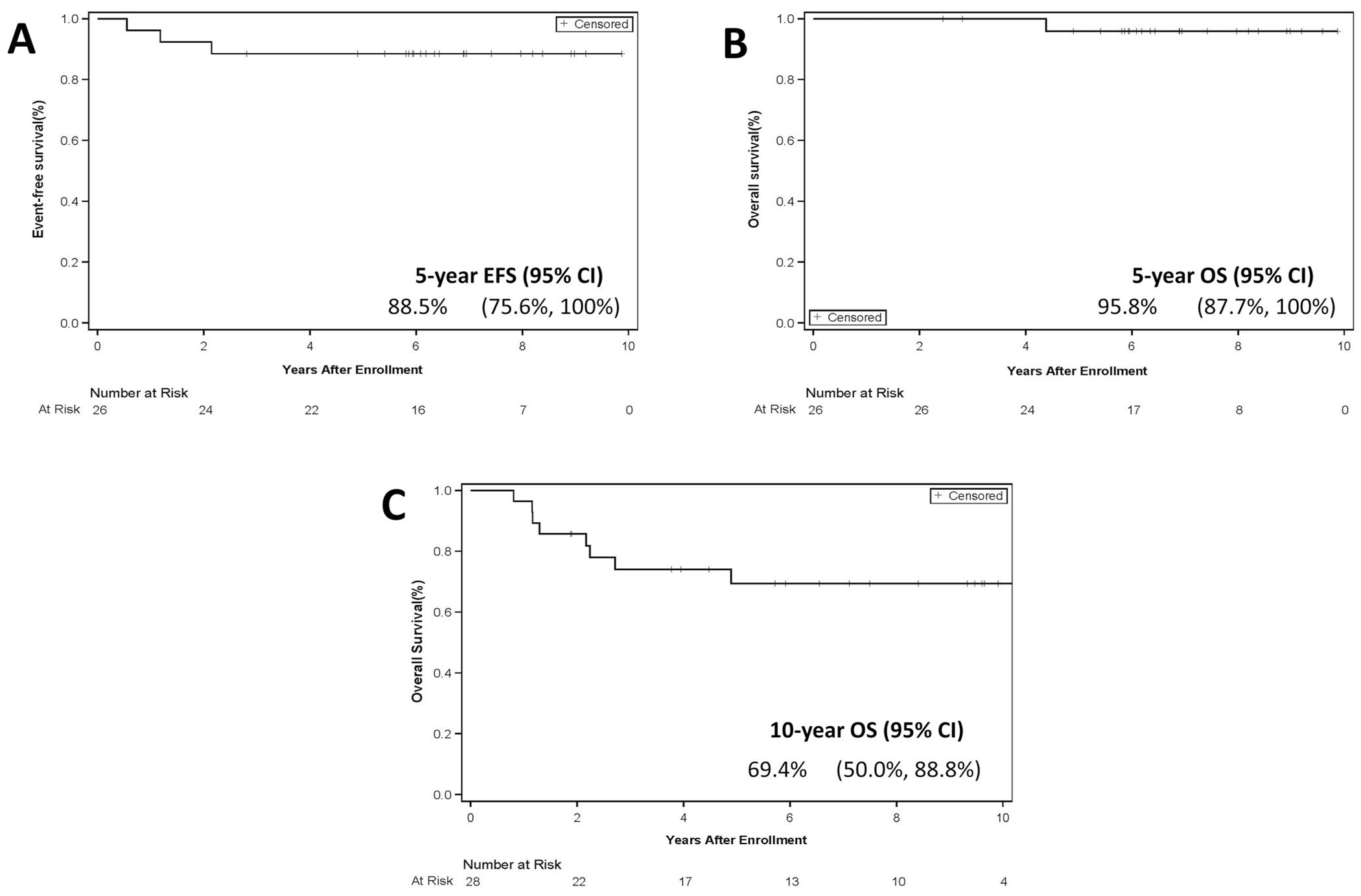
Kaplan–Meier distribution for non-low-risk and recurrent orbital rhabdomyosarcoma. (A) EFS and (B) OS are illustrated for non-low-risk orbital rhabdomyosarcoma, and (C) OS is illustrated for recurrent orbital rhabdomyosarcoma. The estimated 5-year EFS and OS rates for non-low-risk disease and the 10-year OS rate for recurrent disease are indicated at the bottom right in each graph. CI indicates confidence interval; EFS, event-free survival; OS, overall survival.

Twenty-six patients with non–low-risk ORMS were identified, with 25 enrolled on intermediate-risk trials (D9803 and ARST0531) and one enrolled on a high-risk trial (D9802; Table 1). Twenty-one of these patients (80.8%) had alveolar histology, and the majority of patients were group III (*n* = 22; 84.6%). There was one patient with group IV disease. Of the four non-low-risk patients who did not have alveolar histology or group IV disease, one had alveolar histology by institutional review but was classified as *RMS not otherwise specified* on central review, and three patients had alveolar histology at enrollment that was retrospectively reclassified as embryonal based on updated histology definitions (see Materials and Methods, above). Fusion status was available for 13 patients who had non-low-risk ORMS with alveolar histology; eight had a *PAX3-FOXO1* fusion, whereas five were fusion-negative. There were three recurrences; two were local and one was metastatic. With a median follow-up of 6.9 years, the 5-year EFS and OS rates for patients who had non-low-risk ORMS were 88.5% (95% CI, 75.6%-100.0%) and 95.8% (95% CI, 87.7%-100.0%; Figure 2A,B), respectively. All three recurrences developed in patients who were younger than 1.5 years at enrollment (0.2, 1.1, and 1.4 years), and all had localized (one group IIA, two group III), *PAX3-FOXO1* fusion-positive alveolar RMS.

Twenty-eight patients experienced a recurrence, and these occurred at a median of 1.1 years after enrollment (range <0.1 to 4 years). The majority were treated on the low-risk trials D9602 and ARST0331 (*n* = 25; 89.3%) and had embryonal/botryoid histology (*n* = 22; 78.6%; Table 1). Six of these patients (21.4%) did not receive radiation therapy during initial therapy. Twenty-seven recurrences were local, and there was one metastatic recurrence. The single patient with a metastatic recurrence originally had group III, *PAX3-FOXO1* fusion-positive alveolar RMS. With a median follow-up of 9.2 years, the 10-year OS rate from the time of recurrence was 69.4% (95% CI, 50.0%-88.8%; Figure 2C). Treatment approach after recurrence was not collected on these trials.

## DISCUSSION

Our results support a long-term favorable prognosis for patients with low-risk ORMS on COG studies D9602 and ARST0331. Similar to prior reports, our cohort was comprised mainly of embryonal tumors without local invasion (T1) or nodal involvement (N0). It was demonstrated previously that RMS with these features rarely metastasizes and may be properly evaluated with a more limited staging evaluation (for example, omission of bone marrow aspirates/biopsies).^19^ Although the long-term EFS rate for ORMS on D9602 and ARST0331 was <90%, OS remained >95%, and the reduction in cyclophosphamide and radiation doses compared with IRS-IV therapy may reasonably balance survival with potential long-term toxicities. Importantly, our analysis shows that these treatment de-escalations did not result in late recurrences in patients with ORMS. In addition, those with group IIA disease who received a reduced radiation dose (36 Gy) had similarly favorable outcomes, supporting the use of this radiation dose for group IIA ORMS. Patients with non-low-risk ORMS also had excellent 5-year outcomes on intensified therapy on COG intermediate-risk studies D9803 and ARST0531 and high-risk study D9802, demonstrating that intensified therapy for ORMS in patients who have higher risk features is associated with EFS and OS rates similar to those in patients who have low-risk ORMS treated with less intense therapy. Furthermore, a majority of patients with recurrent ORMS may achieve long-term survival, including patients treated on ARST0331 and on non-low-risk trials who received cyclophosphamide in their initial regimen.

The last reported survival outcomes for group I/II ORMS in North America were from IRS-III (3-year FFS of 92% and OS of 100.0% in 25 patients) and IRS-IV (3-year FFS of 89% and OS of 100.0% in 22 patients). Therapy for this small subgroup on both trials included vincristine and dactinomycin, with no radiation for group I and 41.4-Gy radiation for group II.^5^ Although we are unable to make a statistical comparison, the 10-year EFS and OS rates (which included a 36-Gy radiation dose for group IIA) of 88% and 97.6%, respectively, remain favorable.^8^ The substantial portion of patients with tumors initially resected and enrolled as group I/IIA (22.9%; 33 of 197 orbit tumors [16.8%] and 11 of 21 eyelid tumors [52.4%]) is noteworthy given that a biopsy-only approach has been recommended for ORMS on COG trials since D9602. Eleven of the 44 patients in group I/IIA had eyelid disease, and perhaps this superficial location made these tumors more amenable to initial resection. In addition, resected tumors may have been somewhat smaller than nonresected tumors, with 79.5% (35 of 44) of group I/IIA tumors ≤3 cm compared with 63.2% (110 of 174) of tumors in the nonresected cohort, which may have contributed to surgical decision making. The extremely low frequency of group I disease (five of 218 tumors [2.3%]; two of five were eyelid disease) may indicate that achieving up-front negative surgical margins in ORMS is usually not feasible within the limited anatomic space of the orbit.

Other international cooperative groups, including the International Society of Pediatric Oncology Sarcoma Committee (SIOP), the Cooperative Weichteilsarkom Studiengruppe, and the Italian Cooperative Soft Tissue Sarcoma Group (ICG), used chemotherapy regimens similar to those used by the IRSG (including vincristine, dactinomycin, and an alkylating agent) for patients with ORMS but were more likely to withhold radiotherapy for patients who achieved a complete response to chemotherapy. A comparison of outcomes in 306 patients treated from 1978 to 1992 in these cooperative groups showed that the 10-year EFS rate for patients who received radiation was superior to the outcomes of those who did not (82% vs. 53%), with no difference in OS based on cooperative group or use of initial radiotherapy.^18^ A single-institution study of patients with ORMS treated on the ICG RMS-75, SIOP Malignant Mesenchymal Tumor 84 (MMT-84), SIOP MMT-89, SIOP MMT-95, and ICG RMS-05 studies found similar trends in EFS and OS when radiation was not given during initial treatment after a favorable tumor response.^20^ These reports demonstrate a that substantial portion of patients who achieve complete remission with initial chemotherapy may be cured without radiation and that many recurrences after the omission of radiation may be salvaged, preserving excellent OS while sparing some patients the long-term effects of radiation. However, this must be balanced with the burden of treatment for patients with recurrent disease who did not receive radiotherapy, which may include further chemotherapy exposure as well as radiation and/or morbid surgery, such as orbital exenteration.

The precise impact of radiation therapy reductions on toxicity and late effects in low-risk ORMS for group 11A disease (36 vs. 41.4 Gy on IRS-IV) and group III (45 vs. 50.4 or 59.4 Gy on IRS-IV) cannot be determined because late effects of therapy were not collected on D9602 or ARST0331. In a study of proton therapy for adults with orbital/ocular tumors, doses >36 Gy (relative biologic effectiveness) to the cornea were associated with a higher rate of grade 3 chronic toxicity.^21^ In children with group III embryonal ORMS, doses of >40 Gy relative biologic effectiveness to all bones of the orbital rim were associated with significant decreases in orbital volume, affecting facial cosmesis.^22^ In a late-effects study, orbital doses >40 Gy were found to have an increased relative risk of dry eyes compared with doses of 20–40 Gy.^23^ However, a recent COG analysis of ORMS demonstrated that 45 Gy yielded inferior local control when a radiographic complete response was not achieved after 12 weeks of initial chemotherapy.^8^ Therefore, the current low-risk study (ARST2032; ClinicalTrials.gov identifier NCT05304585) includes a dose escalation from 45 to 50.4 Gy in patients with ORMS who have less than a radiographic complete response after 12 weeks.^24^

The only events after 5 years in this analysis were three second malignant neoplasms occurring in two patients. The second malignant neoplasms occurred in the brain, cheek, and tongue near the radiation field. Therapy and cancer-predisposition syndromes likely contributed to their development.^25–28^ The only cancer-predisposition information available for these patients was that there was no personal or family history of neurofibromatosis type I at the time of enrollment. Germline DNA sequencing is not available for the patients in our cohort to identify a specific mutation in the patients with secondary cancers or to determine the rate of cancer-predisposition syndromes in the entire cohort. A recent analysis of 616 blood samples from patients with RMS found germline cancer-predisposition variants in 45 patients (7.3% of the cohort), and these variants were more common in the setting of embryonal histology and younger age.^26^ Implicated predisposition syndromes included Li-Fraumeni, neurofibromatosis type I, DICER1 syndrome, and Costello syndrome, among others.

Patients with non-low-risk ORMS had favorable survival on intermediate-risk and high-risk therapy, with a 5-yearr EFS rate of 88.0% and an OS rate of 95.0%. The majority of these patients (80.8%) had ORMS with alveolar histology. These results compare favorably with outcomes for 24 patients with alveolar ORMS retrospectively analyzed from the IRS I-IV trials, who had a 5-year OS rate of 74.0%.^10^ It is not clear whether the improved outcome is the result of risk-stratified therapy or a significant proportion of patients with fusion-negative alveolar RMS. Of 19 alveolar histology tumors that had fusion testing performed in our study, seven were identified as fusion-negative. Ongoing COG trials are assessing therapy reduction for patients with fusion-negative alveolar RMS.

A substantial portion of recurrent patients with ORMS on COG trials experienced long-term survival. Almost all recurrences were local; the rarity of metastatic recurrence (only one patient of 218 in our cohort) suggests that metastatic surveillance (such as chest imaging) is less valuable in patients who have ORMS compared with surveillance of the primary site. The lack of treatment data after recurrence precludes further comment about optimal treatment in the recurrent setting. It is expected that most underwent orbital exenteration/enucleation, alternate chemotherapy, and possibly radiotherapy. An analysis of orbital sarcomas treated on IRS-III and IRS-IV identified 24 patients with recurrence (21 had RMS). Therapy in this setting included exenteration or enucleation (*n* = 12), reirradiation (*n* = 4), and combination chemotherapy, including the use of etoposide and doxorubicin.^11^ Twenty-two of 24 patients were alive at a median 6.9 years’ follow-up, also demonstrating the high salvage rate of this group of patients. An analysis of 27 patients treated according to the SIOP MMT-95 protocol or the European Pediatric Soft Tissue Sarcoma Group 2005 (EpSSG-2005) protocol confirmed this, with a 5-year EFS rate after first tumoral event of 84.4% (95% CI, 71.5%-98.8%).^29^ Eight of these patients underwent orbital exenteration at first recurrence, and eight of 18 patients who received radiation initially required re-irradiation.

In conclusion, survival outcomes for patients with low-risk ORMS treated on COG studies from 1997 to 2013 were favorable and consistent with prior reports based on shorter follow-up; and patients with non-low-risk ORMS fared well on intermediate-risk and high-risk therapy. Relapsed ORMS appears to be a salvageable disease for a substantial portion of patients, but optimal therapy with consideration of its associated toxicities in the recurrent setting remains to be determined. Further investigation of the differences in therapeutic approaches among international groups on contemporary treatment is warranted. To address this, an international collaborative analysis of ORMS outcomes through the International Soft Tissue Sarcoma Consortium is underway.

## Supplementary Material

supinfo

## Figures and Tables

**TABLE 1:** Demographics, disease characteristics, and survival outcomes for the entire cohort and subgroups of orbital rhabdomyosarcoma.

		ORMS		LR ORMS		Group I/IIA LR		Group I LR		Group IIA LR		NLR ORMS		Recurrent ORMS	
		No.	%	No.	%	No.	%	No.	%	No.	%	No.	%	No.	%
Total patients		218	–	192	–	44	–	5	–	39	–	26	–	28	–
COG trial	D9602	110	50.5	110	57.3	23	52.3	3	60.0	20	51.3	–	–	13	46.4
	D9802	1	0.5	–	–	–	–	–	–	–	–	1	3.9	–	–
	D9803	18	8.3	–	–	–	–	–	–	–	–	18	69.2	2	7.1
	ARST0331	82	37.6	82	42.7	21	47.7	2	40.0	19	48.7	–	–	12	42.9
	ARST0531	7	3.2	–	–	–	–	–	–	–	–	7	26.9	1	3.6
	ARST08P1	0	–	–	–	–	–	–	–	–	–	–	–	–	–
Sex	Male	129	59.2	117	60.9	30	68.2	2	40.0	28	71.8	12	46.2	21	75.0
	Female	89	40.8	75	39.1	14	31.8	3	60.0	11	28.2	14	53.8	7	25.0
Subsite	Orbit	197	90.4	173	90.1	33	75.0	3	60.0	30	76.9	24	92.3	21	75.0
	Eyelid	21	9.6	19	9.9	11	25.0	2	40.0	9	23.1	2	7.7	7	25.0
Age, years	<1	8	3.7	5	2.6	1	2.3	–	–	1	2.6	3	11.5	4	14.3
	1-9	159	72.9	143	74.5	32	72.7	3	60.0	29	74.4	16	61.5	20	71.4
	≥10	51	23.4	44	22.9	11	25.0	2	40.0	9	23.1	7	26.9	4	14.3
Race	Non-White	35	16.1	31	16.1	2	4.6	–	–	2	5.1	4	15.4	8	28.6
	White	168	77.1	147	76.6	39	88.6	3	60.0	36	92.3	21	80.8	19	67.9
	Unknown	15	6.9	14	7.3	3	6.8	2	40.0	1	2.6	1	3.9	1	3.6
Histology	Alveolar	27	12.4	6	3.1	1	2.3	–	–	1	2.6	21	80.8	4	14.3
	Embryonal/Botryoid	169	77.5	166	86.5	40	90.9	4	80.0	36	92.3	3	11.5	22	78.6
	Spindle Cell	14	6.4	14	7.3	3	6.8	1	20.0	2	5.1	–	–	2	7.1
	Other	8	3.7	6	3.1	–	–	–	–	–	–	2	7.7	–	–
Invasiveness	T1	211	96.8	186	96.9	43	97.7	5	100.0	38	97.4	25	96.1	28	100.0
	T2	7	3.2	6	3.1	1	2.3	–	–	1	2.6	1	3.9	–	–
Size, cm	≤5	213	97.7	188	97.9	42	95.5	4	80.0	38	97.4	25	96.1	27	96.4
	>5	5	2.3	4	2.1	2	4.6	1	20.0	1	2.6	1	3.9	1	3.6
Regional nodes, clinical	N0	215	98.6	190	99.0	44	100.0	5	100.0	39	100.0	25	96.1	28	100.0
	N1	1	0.5	–	–	–	–	–	–	–	–	1	3.9	–	–
	Nx	2	0.9	2	1.0	–	–	–	–	–	–	–	–	–	–
Fusion status	*PAX3-FOXO1*	11	5.1	3	3.2	–	–	–	–	–	–	8	30.8	4	14.3
	*PAX7-FOXO1*	2	0.9	2	1.0	–	–	–	–	–	–	–	–	–	–
	Negative Fusion	13	6.0	6	3.1	1	2.3	–	–	1	2.6	7	26.9	–	–
	Unknown	192	88.1	181	94.3	43	97.7	5	100.0	38	97.4	11	42.3	24	85.7
Clinical group	Group I	5	2.3	5	2.6	5	11.4	5	100.0	–	–	–	–	1	3.6
	Group II (all IIA)	42	19.3	39	20.3	39	88.6	–	–	39	100.0	3	11.5	5	17.9
	Group III	170	78.0	148	77.1	–	–	–	–	–	–	22	84.6	22	78.6
	Group IV	1	0.5	–	–	–	–	–	–	–	–	1	3.9	–	–
Recurrence	None	190	87.2	167	87.0	39	88.6	4	80.0	35	89.7	23	88.5	–	–
	Local	27	12.4	25	13.0	5	11.4	1	20.0	4	10.3	2	7.7	–	–
	Metastatic	1	0.5	–	–	–	–	–	–	–	–	1	3.9	–	–
10-year EFS/5-year EFS for NLR (95% CI), %				85.5 (77.0–94.0)		88.0 (72.6–100.0)		80.0 (39.5–100.0)		89.1 (72.4–100.0)		88.5 (75.6–100.0)			
10-year OS/5-year OS for NLR (95% CI), %				95.6 (90.8–100.0)		97.6 (90.0–100.0)		100.0 (100.0–100.0)		97.3 (88.3–100.0)		95.8 (87.7–100.0)		69.4 (50.0–88.8)	

Abbreviations: COG, Children’s Oncology Group; EFS, event-free survival; LR; low-risk; NLR, non-low-risk; ORMS, orbital rhabdomyosarcoma; OS, overall survival.
