# Supplementary material for: Survival of patients with orbital and eyelid rhabdomyosarcoma treated on Children’s Oncology Group studies from 1997 to 2013: A report from the Children’s Oncology Group
Source: Cancer. Author manuscript; Available in PMC 2024 Jun 1. (PMC10288338; doi:10.1002/cncr.34723)
Supplement: supinfo [file NIHMS1905856-supplement-supinfo.docx]

**Table S1:** COG rhabdomyosarcoma trials from 1997-2013 included in this analysis. Abbreviations: NCT#: National Clinical Trial identifier number, ORMS: orbital rhabdomyosarcoma, VCR: Vincristine, DAC: Dactinomycin, CYC: Cyclophosphamide, IRI: Irinotecan, TOP: Topotecan, DOX: Doxorubicin, ETO: Etoposide, IFO: Ifosfamide, TEM: Temozolomide, CIX: Cixutumumab.

| **Study** | **NCT#** | **Years** | **Risk Group** | **Study Strategy** | **Chemotherapy for ORMS Patients** |
| --- | --- | --- | --- | --- | --- |
| **D9602** | NCT00002995 | 1997-2004 | Low | Elimination of CYC for Group III ORMS, radiation reduction | VCR, DAC |
| **D9802** | NCT00003955 | 1999-2004 | High | Incorporation of VCR/IRI | VCR, DAC, CYC, +/- VCR, IRI |
| **D9803** | NCT00003958 | 1999-2005 | Intermediate | Incorporation of TOP/CYC, response-adjusted radiotherapy | VCR, DAC, CYC, +/- TOP, CYC |
| **ARST0331** | NCT00075582 | 2004-2010 | Low | Reduce length of therapy, lower CYC dose, radiation reduction | VCR, DAC, CYC for 12 weeks, then VCR, DAC for 12 weeks |
| **ARST0431** | NCT00354744 | 2006-2008 | High | Multiagent chemotherapy with dose interval compression | VCR, IRI, DOX, CYC, ETO, IFO, DAC |
| **ARST0531** | NCT00354835 | 2006-2012 | Intermediate | Incorporation of VCR/IRI, early radiotherapy | VCR, DAC, CYC, +/- VCR, IRI |
| **ARST08P1** | NCT01055314 | 2010-2013 | High | Incorporation of TEM, CIX to ARST0431 therapy | VCR, IRI, DOX, CYC, ETO, IFO, DAC, + TEM or CIX |
